# MiR-34a promotes DCs development and inhibits their function on T cell activation by targeting WNT1

**DOI:** 10.18632/oncotarget.15228

**Published:** 2017-02-09

**Authors:** Anfei Huang, Yi Yang, Si Chen, Fei Xia, Di Sun, Deyu Fang, Sidong Xiong, Liping Jin, Jinping Zhang

**Affiliations:** ^1^ Institutes of Biology and Medical Sciences, Soochow University, Suzhou, Jiangsu Province 215123, People’s Republic of China; ^2^ Department of Pathology, Northwestern University Feinberg School of Medicine, Chicago, IL 60611, USA; ^3^ Clinical and Translational Research Center, Shanghai First Maternity and Infant Hospital, Tongji University School of Medicine, Shanghai 200040, China

**Keywords:** miR-34a, cDCs, development, IL-17a, T cell activation

## Abstract

MicroRNAs serve important functions in numerous biological processes. Whether microRNAs also act on dendritic cell (DC) differentiation and function remains unclear. In this study, both conventional DCs (cDCs) and plasmacytoid DCs (pDCs) were increased in miR-34a overexpressing bone marrow chimeric and transgenic (TG) mice. Further experiments showed that miR-34a promoted preDC differentiated into cDCs and pDCs without affecting the proliferation and apoptosis of DCs. Luciferase report assay and Western blot experiments demonstrated that WNT1 is the direct target of miR-34a in DCs. Interestingly, miR-34a overexpressing cDCs also produced a large amount of IL-17a and suppressed T cell activation because of the inhibition of TCF1 expression, thus increasing RORγT expression. Taken together, miR-34a promotes preDC to differentiate into cDCs and pDCs, as well as inhibits the function of cDCs on the activation of CD4^+^ T cells by producing IL-17a.

## INTRODUCTION

Dendritic cells (DCs) derived from hematopoietic stem cells play crucial roles in innate and adaptive immune response and in maintaining immune tolerance to self-tissues [1]. DCs are a heterogeneous group of cells that have been divided into different subsets with distinct patterns of cell-surface molecule expression, which also have distinct life span and immune functions [2, 3]. Two major subsets of DCs in mice and humans are plasmacytoid DCs (pDCs) and conventional DCs (cDCs); the latter is further divided into CD8^+^ and CD8^-^ subsets [4]. Weissman et al. (2000) showed that both CD8^+^ and CD8^-^ DCs can arise from clonogenic common myeloid progenitors in both thymus and spleen. The report ends the disputation that CD8^+^ and CD8^-^ DCs arise from lymphoid and myeloid hematopoietic progenitors, respectively [5]. Recent studies show that DC development progresses from the MDP (macrophage/DC precursor) to common DC precursors (CDP) that give rise to pDCs and cDCs, but not monocytes. Unlike monocytes and pDCs, cDCs in lymphoid tissues are believed to emerge from the bone marrow as immature cells called pre-cDCs produced by CDP. Pre-cDCs enter lymph nodes through high endothelial venules and later disperse and integrate into the DC network [6–8]. Although the general pathway of DC development is obtained, the molecular regulations involved in this pathway remain unclear.

Based on these findings, most of the progress in this field focused on the functions of transcriptional factors and cytokines, among which FMS-related tyrosine kinase 3 ligand (FLT3L), M-CSF, and GM-CSF are important for DC development [9]. Little is known about the transcriptional program that controls DC lineage commitment in immature progenitors with PU.1, Ikaros, and GFI1, which are likely the prime candidates for DC-specific factors [9]. PU.1 can regulate GM-CSFR and FLT3 expression and is essential for FLT3-L and GM-CSF-containing culture systems *in vitro*. Interestingly, FLT3 signaling can activate PU.1 expression in MEP, which suggests a self-enforcing loop between FLT3 and PU.1 in DCs [10, 11]. Deficiency of Ikaros results in the absence of most DCs; a hypomorphic mutation of Ikaros results in a specific loss of pDCs [12]. GFI1 is expressed in DC precursors, and GFI1-deficient hematopoietic progenitor cells are incapable of developing into DCs *in vitro* in the presence of either FLT3L or GM-CSF and instead differentiate into macrophages [13]. This occurrence suggests that GFI1 is a crucial modulator of DC versus macrophage development.

MicroRNAs (miRNAs) are small non-coding RNAs that post-transcriptionally regulate gene expression by binding to their target mRNAs, thereby inhibiting their translation or affecting their stabilities. More than 60% of all human coding genes are predicted to contain miRNA binding sites in their 3′ untranslated region. Therefore, miRNAs are key regulators in many biology processes [14]. Many studies have already demonstrated that miRNAs are involved in the modulation of hematopoietic lineage commitment such as in T cells, B cells and NK cells commitment [15–21]. Some miRNAs are also functional in DC development and activation. Treatment of monocytes with miR-21 or miR-34a inhibitors partially stalls MDDC (Myeloid derived dendritic cell) differentiation [22]. miR-221 and miR-222 are more highly expressed in cDCs than in pDCs. Inhibition of function of either of these two miRNAs in BM cells by using their agonist oligo increased pDC/cDC ratio, which suggests that mir-221/mir222 may promote pDC development [23]. However, the manner by which microRNAs regulate DC development remains largely unclear.

In this study, we first establish the miR-34a overexpressing bone marrow chimeric and transgenic (TG) mouse model. Through FACS analysis, we found that the number of cDCs and that of pDCs increased. Further study suggested that miR-34a can promote preDCs to develop into pDC and cDC but did not affect the apoptosis and proliferation of pDCs or cDCs. Luciferase report assay and Western blot experiments demonstrated that WNT1 was a direct target of miR-34a in DC cells. Further experiments demonstrated that miR-34a significantly affected DC function. MiR-34a-overexpressing DCs produced dramatically high level of IL-17a without changes in their activation and capability for antigen-uptake. IL-17a was fed back on DCs, thus impairing the activation of CD4^+^ T cells, possibly by influencing the Wnt1/TCF/RORγt pathway in DCs. In sum, miR-34a promotes both pDC and cDC development, as well as inhibits the function of DCs on CD4^+^ T cell activation.

## RESULTS

### Overexpression of miR-34a promotes the development of cDCs and pDCs

**Figure 1:**
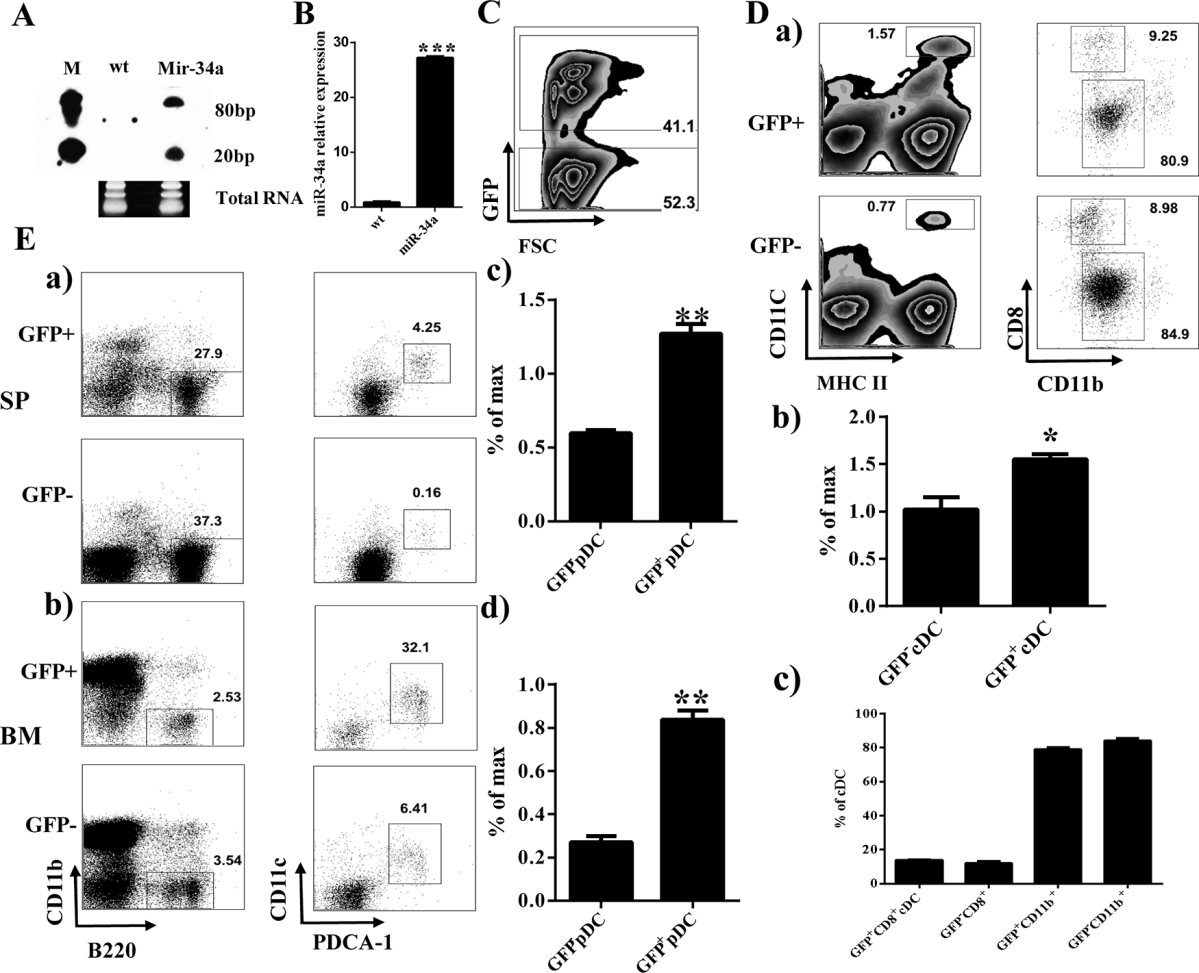
Overexpression of miR-34a increases percentage of cDCs and pDCs **A, B**. Expression of miR-34a in 293T (wt) and 293T transfected with pMDH-PGK-GFP-miR-34a plasmid (Mir-34a) detected by Northern blot (*A*) and real time PCR (*B*). **C**. Expression of GFP in BM from miR-34a Chimera mice. **D**. Percentages of CD11c^+^MHC II^+^ cDC (*a left, b*) and their subset CD8^+^CD11b^-^, CD8^-^CD11b^+^ (*a right, c*). **E**. Percentage of pDC (CD11b^-^B220^+^CD11c^+^PDCA-1^+^) in spleen (*a, c*) and BM (*b, d*). * means p<0.05, ** means p<0.01, ***means p<0.001. The data represent 5 repeats.

To assess the role of miR-34a on hematopoiesis, we initially constructed the miR-34a overexpressing retrovirus plasmid pMDH1-PGK-GFP-mir-34a. The encoding efficiency of this plasmid was confirmed by real time PCR and Northern blot (Figure 1A and 1B). Thus, we infected mouse HSC/progenitor enriched BM cells with miR-34a-expressing viruses. These miR-34a overexpressing BM cells were transplanted into lethally irradiated recipients. Flow cytometry analysis of the BM and spleen in stably engrafted primary chimera mice showed increasing numbers and frequency of cDCs and pDCs 8 weeks after transplantation (Figure 1C, 1D and 1E). Additionally, we also detected the expression of miR-34a in several DC progenitors including GMPs, CMPs, Pro-DCs and Pre-DCs. Interestingly, miR-34a was highly expressed in Pre-DCs (Supplementary Figure 1).

**Figure 2:**
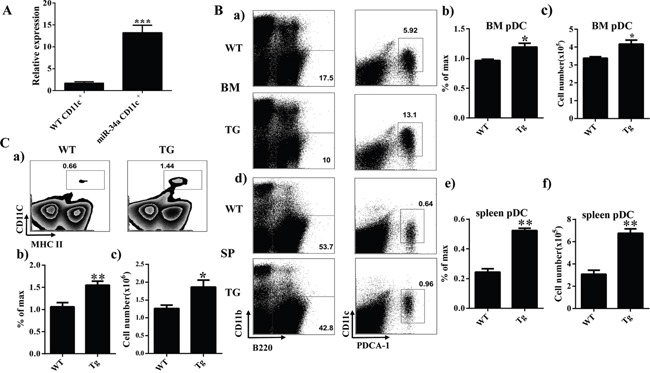
Overexpression of miR-34a increases number of cDC and pDC in miR-34a transgenic mice **A**. Expression of miR-34a in wild type (wt) and miR-34a TG mice CD11c^+^ cells (miR-34a) detected by real time PCR. **B**. Percentage and absolute number of pDC in BM (*a, b, c*) and spleen (*d, e, f*) in miR-34a TG and WT mice. **C**. Percentage (*a, b*) and absolute number (*c*) of cDC in spleen in WT and miR-34a mice. * means p<0.05, ** means p<0.01, ***means p<0.001. The data represent 5 repeats.

To verify the phenotype of miR-34a chimera mice further, we established a miR-34a transgenic (TG) mouse model. Quantitative PCR indicated that the expression of miR-34a was dramatically increased in CD11c^+^ cells from miR-34a TG mice (Figure 2A). FACS analysis of BM and spleen from miR-34a-TG mice showed similar phenotypes as those in miR-34a chimera mice (Figure 2). These data suggest that miR-34a is involved in the development of pDCs (Figure 2B) and cDCs (Figure 2C). To extensively investigate the role of miR-34a in DC development, we also examined the frequency of DCs in miR-34a knockout mice. In agreement with the phenotype in miR-34a transgenic mice, miR-34a deletion significantly impaired cDCs development and slightly affected pDCs development (Supplementary Figure 2). Thereafter, we focused on the function of miR-34a on cDC and pDC development.

### miR-34a does not affect the apoptosis and proliferation of cDCs and pDCs

To elucidate how miR-34a prompted cDC and pDC development, we first evaluated the apoptotic rate of cDC and pDC from transgenic mice. FACS analysis showed a comparable apoptotic cDCs (Supplementary Figure 3A and 3B) and pDCs (Supplementary Figure 3C-3F) between wild-type (WT) and TG mice. To address whether the improvement of cDCs and pDCs was attributed to miR-34a, the proliferation of cDCs and pDCs was subjected to flow cytometry analysis by *in vivo* BrdU incorporation assay. The percentage of BrdU positive cells among splenic cDCs (Supplementary Figure 4A and 4B) or pDCs from BM or spleen (Supplementary Figure 4C-4F) also exhibited no significant difference between WT and miR-34a-TG mouse, which suggests that miR-34a also did not affect the proliferation or self-renewal of both cDCs and pDC *in vivo*. These observations demonstrate that the increase in cDCs and pDCs in miR-34a TG mice is not caused by impairing cell apoptosis and proliferation.

### miR-34a has function in the checkpoint of DC development during which pre-DCs differentiate into cDCs/pDCs

**Figure 3:**
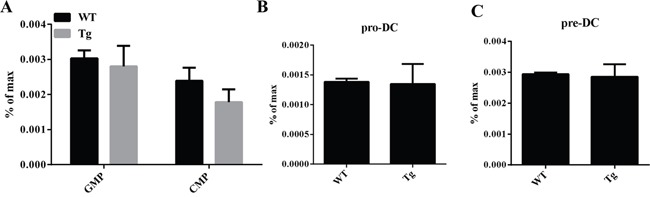
GMP, CMP, ProDC, and PreDCs in miR-34a transgenic mice **A**. Percentage of GMP (Lin^-^C-kit^+^Sca1^-^CD16/32^+^CD34^+^) and CMP (Lin^-^C-kit^+^Sca1^-^CD16/32^-^CD34^+^) in WT and miR-34a mice BM (*a, b*). **B**. Percentage of proDC (Lin^-^IAb^-^CD11c^-^flt3^+^C-kit^+^) in BM in WT and miR-34a transgenic mice (*a, b*). **C**. Percentage of preDC (Lin^-^CD11c^+^IAb^-^ flt3^+^Sirpa^+^) in BM in WT and miR-34a transgenic mice (*a, b*). The data represent 3 repeats.

Overexpression of miR-34a does not impair cDC/pDC apoptosis and proliferation. Thus, we try to investigate whether this increase of cDC/pDCs can be attributed to the fact that miR-34a promotes DC development. First, we examined the frequency of DC progenitors by flow cytometry. The results show that the percentages of these subsets, including GMP/CMP (Figure 3A), Pro-DC (Figure 3B) and Pre-DC (Figure 3C), of miR-34a TG mice were similar to those of WT mice. Additionally, we ask whether miR-34a intrinsically promotes pre-DCs proliferation, we detect the proliferation activity of Pre-DCs *in vivo*, FACS data indicates miR-34a does not affect Pre-DCs expansion *in vivo* (Supplementary Figure 5). Taken together, we conclude that miR-34a is involved in promoting the process of preDC development into cDC.

### WNT1 is the functional target of miR-34a in DCs

**Figure 4:**
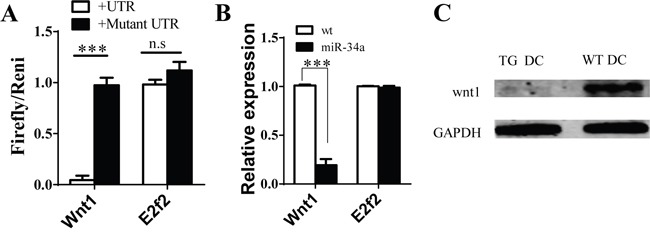
Wnt1 as target of miR-34a in DCs **A**. Luciferase report assay for verifying miR-34a target Wnt1 3′UTR but not E2F2 and their mutated 3′ UTR. **B**. Expression of Wnt1 and E2F2 genes in cDC from WT and miR-34a transgenic mice detected by real time PCR. **C**. Expression of WNT1 protein in cDC in WT (WT DC) and mir-34a TG (TG DC) mice. GAPDH as loading reference. ***means p<0.001. The data represent 5 repeats.

To explore the molecular mechanism of miR-34a in DC development, we first searched for the predicted targets of miR-34a by using online software. We notified some molecules, such as E2F2 and WNT1, which may contribute to DC development, acted as the predicted targets of miR-34a. To validate whether miR-34a directly recognizes the 3′-UTR of these transcripts, we cloned oligonucleotides representing the presumed target sites into the 3′-UTR of the luciferase gene. We also constructed the corresponding reporter vectors containing mutated binding sites. Luciferase activity was examined in 293T cells co-transfected with one of these constructs, and vector expressing miR-34a and TK plasmid. The relative activity of luciferase indicates that E2F2 is not a miR-34a target (Figure 4A). In contrast, miR-34a reduced the luciferase activity of the reporter construct containing miR-34a binding site in WNT1 3′-UTR, whereas no effect was observed for construct containing a mutated WNT1 seed site (Figure 4A). These findings suggest that WNT1 is the target of miR-34a. We also found that the amount of WNT1 mRNA decreased based on RT-PCR assay (Figure 4B), and the expression of WNT1 protein was also blocked in CD11c^+^ cells purified from spleen of miR-34a-TG mice measured by Western blot (Figure 4C). These observations suggest that miR-34a suppresses the expression of WNT1.

### Overexpression of miR-34a in cDC inhibits CD4^+^ T cell activation

**Figure 5:**
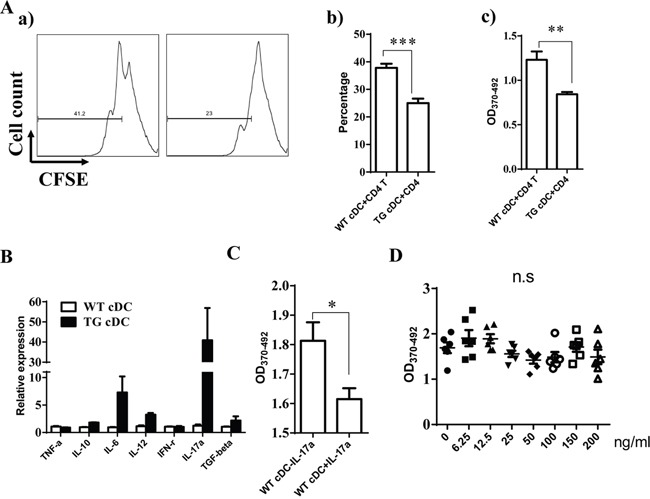
miR-34a overexpressing cDC inhibits CD4+ T cell activation **A**. CD4^+^ T cells from OVA-immunized mice were activated with OVA pre-loaded cDCs from WT or miR-34a transgenic mice, proliferation were evaluated by CFSE labeling assay (*a, b*) or BrdU ELISA (*c*). **B**. Expression of some of cytokines in cDC from miR-34a TG mice (black) and WT mice (blank). **C**. Addition of IL-17a inhibits proliferation of CD4^+^ T cells in WT cDCs plus CD4^+^ T cells culture system. **D**. Direct addition of IL-17a in CD4^+^ T cells without DCs does not affect CD4^+^ T cell activation. * means p<0.05, ** means p<0.01, ***means p<0.001. The data represent 5 repeats.

Given that DCs serve a critical function on T cell response, we further explore whether miR-34a overexpression in DCs impairs DC function on T cell response. First, we incubated WT cDCs with OVA overnight and then checked miR-34a expression using RT-PCR. As expected, miR-34a expression was down-regulated in OVA-incubated cDCs compared to naive cDCs (Supplementary Figure 6A), which suggests that miR-34a may serve as a critical functional role in antigen-presentation or maturation of DCs. To test whether miR-34a indeed affects the antigen-presentation of DCs, we checked the uptake of FITC-labeled OVA protein by cDCs from WT or miR-34a TG mice at 2, 4, and 6 h using FACS (Supplementary Figure 6B, a and b). We found that the up-taken OVA in cDCs from two groups of mice were nearly identical, which suggests that miR-34a overexpression did not affect the antigen-uptake of DCs. Moreover, we also detected the level of maturation markers of DCs including CD80 and CD86. Interestingly, cDCs from WT and TG mouse showed a comparable expression of CD80, CD86 (Supplementary Figure 7). These results suggest that miR-34a did not affect the activation of cDCs. To extensively investigate whether mir-34a-overexpressing cDCs impaire CD4 T cells activation, OVA-immunized CD4^+^ T cells labeled with CFSE were co-cultured for 72h with cDCs purified from WT or miR-34a-TG mice. Excitingly, We notified that the proliferation of CD4^+^ T cells was dramatically suppressed in the miR-34a-TG group by flow cytometry analysis or BrdU incorporation ELISA assay (Figure 5A), which suggests that miR-34a overexpression in cDCs impair the response of CD4^+^ T cells. To further explore how miR-34a transgenic cDCs affect T cell response, we subsequently measured the expression profile of cytokines in cDCs by using RT-PCR. Surprisingly, IL-17a was significantly highly expressed in cDCs from miR-34a TG mice (Figure 5B). Intracellular staining also demonstrated that IL-17a was highly expressed in miR-34a overexpressing cDCs (Supplementary Figure 8). Furthermore, the addition of IL-17a into co-culture system of wild type cDCs and T cells can also suppress the proliferation of CD4^+^ T cells (Figure 5C). These results strongly suggest that miR-34a overexpression in cDCs can inhibit CD4^+^ T cell response, which may be mediated by IL-17a.

**Figure 6:**
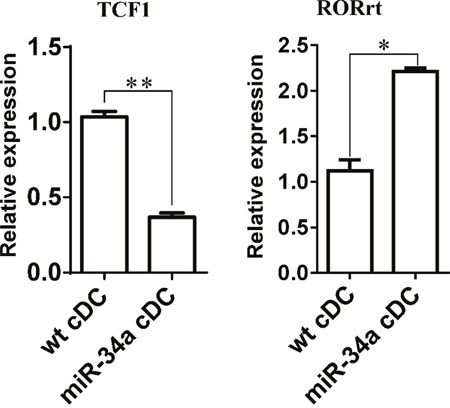
Expression of TCF1 and RORrt in miR-34a overexpressed DCs Expression of TCF1 and RORrt genes in WT cDCs and miR-34a overexpressed cDCs were detected by real time PCR. * means p<0.05, ** means p<0.01. The data represent 3 repeats.

IL-17a may affect the response of CD4^+^ T cells directly or indirectly using DCs as a mediator. Thus, we first assessed the expression of IL-17 receptor in cDCs and CD4^+^ T cells to distinguish these two possibilities. The results also showed that IL-17 receptor expression was significantly higher in cDCs than in CD4^+^ T cells (Supplementary Figure 9). When we stimulated CD4^+^ T cells with anti-CD3/CD28 antibodies in the absence of DCs, we did not observe any inhibition after the addition of different doses of IL-17a (Figure 5D). These findings suggest that IL-17a may directly act on cDCs and exert an inhibition function on CD4^+^ T cell activation via DCs. Some evidence has already shown that WNT1 can modulate TCF1 to regulate RORγT expression, thus regulating IL-17 transcription [24, 25]. We thus detected the expression of TCF1 and RORγT in cDC from miR-34a-TG mice. Consistent with our expectations, RT-PCR results showed that TCF1 expression decreased, but that of RORγT significantly increased in cDCs from miR-34a-TG mice compared with that in their WT counterpart (Figure 6). In sum, miR-34a overexpression in DCs downregulates the WNT1 expression, thus resulting in the downregulation of TCF1, upregulation of RORγT and consequent upregulation of IL-17a, which affects cDCs in turn, as well as the inhibition of CD4^+^ T cell activation via DCs.

## DISCUSSION

Accumulating evidence reveals that microRNAs serve very important functions in lymphocyte development [26, 27]. MicroRNAs regulate DC activation (let-7i, miR-142-3p, miR-146a, the miR-148 family, and miR-155), as well as cytokine production and development of DCs (miR-155) [28, 29]. In 2009, by using *in vitro* culture system, Hashimi et al. found that miR-21 and miR-34a are necessary for human DC differentiation from monocytes [22]. To explore the function of miR-34a on DC development and function further, we adopted miR-34a overexpressing chimera and miR-34a TG mouse models in this study. FACS analysis showed that overexpressing miR-34a alone can result in an increase in the percentage and absolute number of cDCs and pDCs, both in the spleen and BM of chimeric and TG mice. We further investigated how miR-34a overexpression in hematopoietic system induces these increases in cDCs and pDCs. We first investigated whether miR-34a affects apoptosis and proliferation of DCs by Annexin V staining and BrdU incorporation analysis, respectively. Results showed that the apoptosis and proliferation of miR-34a overexpressing cDCs and pDCs were similar to their WT counterparts. These findings suggest that miR-34a does not affect the apoptosis or proliferation of cDCs and pDCs. Ruling out these two possibilities, we further validated the last one, which is whether acceleration of development from progenitors into DCs in miR-34a-overexpressing mice results in an increased number of DCs. To verify this idea, we analyzed the percentages of progenitor cells upstream and found that GMP, CMP, PreDC, and ProDC exhibited no difference between miR-34a-overexpressing and WT mice. But preDC cultured *in vitro* from mir-34a TG mice could produce more DCs than PreDC from WT mice. Thus, we assumed that miR-34a may push preDC to develop into cDCs and pDCs.

To determine the targets of miR-34a, we searched for potential miR-34a targets using computer-based predictions from mirwalk and obtained molecules associated with DC development through Gene Ontology analysis. We then overlap these two groups of molecules to determine the common ones. Finally, we selected E2F2 and WNT1 as candidates for miR-34a target. Luciferase assay revealed that 3′UTR of WNT1 mRNA can be an efficient binding site of miR-34a, but not of E2F2. Western blot further revealed that WNT1 protein expression largely decreased in miR-34a cDCs. These data strongly suggest that WNT1 is the direct target of miR-34a in DCs. The most important experiment in microRNA research is to seek the functional target of microRNA. We thus preserved the expression of WNT1 in miR-34a-TG mice based on a vector containing WNT1 full-length coding sequence without 3′UTR to investigate whether WNT1 is the functional target. FACS analysis showed that overexpression of WNT1 can decrease the numbers of cDCs and pDCs in miR-34a-TG mice. These results demonstrate that WNT1 is the functional target of miR-34a in DCs.

Accumulating evidence suggests that WNTs play important roles in immune system. Ablation of WNT3a inhibited the differentiation potential of CMPs and GMPs. Activation of WNT3a inhibits B and pDC differentiation. Addition of WNT3a can promote the CD11c^+^ DC development but decrease the Gr1^+^CD11b^+^ immature myeloid cells development. Meanwhile, activation of WNT3a can enhance the T cell proliferation [30–33]. Our current study showed that downregulating WNT1 by miR-34a can promote cDC and pDC development, which suggests that WNT1 inhibits the PreDC to develop into cDCs and pDCs.

DCs are the most important antigen-presenting cells. Once they uptake the antigen, DCs stabilize peptide-loaded MHC molecules on their surfaces and start to secrete cytokines that polarize adaptive immune responses to enable effective targeting of a specific pathogen. Some studies have already shown that microRNAs serve an important function in these processes. Although miR-155 does not affect DC development because the numbers of CD8a^+^ cDCs, CD8a^-^ cDCs, and pDCs are not altered in mice lacking miR-155, it appears to be involved in the maturation of DCs. Deficiency of miR-155 significantly decreases levels of MHC II, CD40, and CD86 on DC surface, accompanied by low levels of secreted IL-12p40, IL-12p35, and TNF-α, thus impairing the capability of DCs to activate T cells [34]. Members of the miR-148 family (miR-148a, miR-148b, and miR-152) are upregulated upon DC stimulation by LPS, and their overexpression reduces MHC II expression on DC surface, inhibits the secretion of some proinflammatory cytokines, and reduces DC-mediated CD4^+^ T cell expansion. Correspondingly, MHC II expression, cytokine production, and CD4^+^ T cell proliferation increase when LPS-stimulated DCs are treated with miR-148 family inhibitors [35]. In this study, miR-34a was more highly expressed in immature DCs than in antigen-stimulated DCs, which suggests that miR-34a may serve a function in DC maturation. However, we did not find the expressions of some activation markers, such as CD80, CD86, and MHCII, to be significantly different between WT DCs and miR-34a overexpressing DCs. We also did not find any alteration in the capability for antigen-uptake of miR-34a overexpressing DCs compared with WT DCs. However, miR-34a-overexpressing DCs can impair the antigen-specific proliferation of CD4^+^ T cells. Cytokine expression detected by RT-PCR showed that IL-17a expression was dramatically enhanced in miR-34a overexpressing DCs. The addition of IL-17 to the co-cultured system of DCs and CD4^+^ T cell can inhibit the CD4^+^ T cell proliferation. Neutralization of IL-17 in this co-cultured system can recover the CD4+ T cell proliferation (data not shown). High expression of miR-34a in immature DC may contribute to the self-antigen loaded DCs suppress the auto-immune response T cells response and maintain homeostasis. However, the detail mechanism remain explore in the furture.

Ample evidence suggests that WNT/β-catenin/TCF1 pathway regulates T cell function in both the central and peripheral immune system [36]. In the thymus, regulation of T cell proliferation, survival, and apoptosis are required at multiple stages of T cell development. In peripheral lymphoid tissues, TCF-1 regulates the differentiation of naïve CD4^+^ T cells into Th1/Th2/Th17 cells that are competent to mediate immune response [36]. However, no study suggests that this WNT/β-catenin/TCF1 pathway also serves a function in DCs. To explain why miR-34a overexpression in DCs can increase the expression of IL-17a, we checked TCF1 and RORγT expression in miR-34a overexpressing DCs. As predicted, TCF1 was downregulated, whereas RORγT was upregulated in miR-34a overexpressing DCs. These findings suggest that WNT1/β-catenin/TCF1 pathway also functioned in DCs and regulated the expression of IL-17a. IL-17a secreted by DCs acts on DCs in turn, thus inhibiting T cell response. This research is the first to elaborate on the involvement of the miR-34a-WNT1/TCF1 pathway in DC control on T cell response. Further studies will be required to determine whether IL-17-secreted dendritic cells are a specific population and how IL-17 reactions to DCs affect the impairment of T cell response.

## MATERIALS AND METHODS

### Mice

Six- to eight-week-old C57/BL6 mice and SJL mice were purchased from Shanghai SLAC Laboratory Animal Co. The miR-34a transgenic mice used in this study were bred by Cyagen Bioscience, Inc. as described below. miR-34a KO mice were purchased from Animal Research Institute of Nanjing University. All mice were maintained in a barrier facility at Soochow University. All animal experiments were approved by the Institutional Animal Care and Use Committee of Soochow University.

### Antibodies

The following antibodies were purchased from Biolegend Inc.: anti-CD3-pacific blue (17A2), anti-CD4-pacific blue (GK1.5), anti-CD4-FITC (GK1.5), anti-CD8-pacific blue (53-6.7), anti-CD8-APC (53-6.7), anti-NK1.1-pacific blue (PK136), anti-CD11b-pacific blue (M1/70), anti-CD11c-pacific blue (N418), anti-CD11c-percp-cy5.5 (N418), anti-I-Ab-FITC (KH74), anti-I-Ab-PE (KH74), anti-CD34-PE (MEC14.7), anti-B220-APC-Cy7 (RA3-61B2), anti-B220-pacific blue (RA3-61B2), anti-CD117-PE-Cy7 (2B8), anti-PDCA1-APC (927), anti-CD135-APC (A2F10), anti-CD172-biotin (P84), anti-IL-17a-PE (TC11-18H10.1), and strepedavidin-PE-Cy7 (Biolegend, CA). The anti-WNT1 antibody was purchased from affinity Bioscience (Affinity, OH).

### Preparation of chimera and TG mice models

A fragment of miR-34a gene was polymerase chain reaction (PCR)-amplified from mouse genomic DNA using the following primers: F: 5′-CCG CTC GAG CCC CCT TGT GGA AGC CGG AAG GCA T-3′; R: 5′-CCG GAA TTC TTT TTA AGT GCT CAC ACT CCA GAC C-3′. The resultant fragment was cloned into the XhoI and EcoRI sites in the vector of pMDH1-PGK-GFP2.0, and the insert was confirmed by sequencing. Retroviral supernatant was generated by using standard procedures after calcium phosphate transfection of pMDH1-PGK-GFP-miR-34a and pCL-ECO viral packaging construct into 293T cells. To enrich hematopoietic stem/progenitor cells, donor mice were injected i.p. with 5 mg of 5-fluorouracil 5 d before BM harvest. BM cells were collected by flushing the tibia and femur with PBS/1% FBS, and red blood cells were lysed with ACK lysis buffer (8.29 g NH4Cl, 1 g KHCO_3_, 37.2 mg EDTA in 1L H2O, pH 7.4). C57/BL6 normal mice BM cells were infected with retrovirus as in a previously described protocol [37]. Infected cells were resuspended in PBS and then injected (i.v.) into lethally irradiated (8.5 Gy) recipient mice to establish miR-34a chimera mice. After two months to four months, the mice were sacrificed, and some immune cells were harvested for use in the relative experiments.

Meanwhile, pMDH1-PGK-GFP2.0-miR-34a plasmids were linearized by HindIII restriction enzyme digestion. Linearized plasmids were micro-injected into ES cells with C57/BL6 background. The ES cells were then implanted into pseudocyesis C57/BL6 mice (Cyagen Biosciences Inc. Guangzhou). Positive offspring mice were bred with other mice from the same founder. Six- to eight-week-old miR-34a-TG mice were used for relative experiments.

### Cell staining and flow cytometry

Mouse BM and spleen cells were stained with anti-CD11c, anti-IAb, anti-CD8, anti-CD11b, and anti-PDCA1 antibodies to detect the percentages of cDCs and pDCs, BM cells were also stained for preDC, proDC, GMP, and CMP analysis with anti-CD11c, anti-IAb, anti-Flt3, anti-sirpα, anti-c-kit, anti-Sca1, and Lineage cocktail antibodies (anti-CD3, anti-CD4, anti-CD8, anti-B220, anti-Gr1, anti-CD11b, anti-NK1.1, anti-Ter119). For apoptosis assay, BM or spleen cells were first stained with anti-CD11c, anti-IAb, anti-CD8, anti-CD11b, and anti-PDCA1 antibodies. The cells were then washed and stained with annexin V in annexin V binding buffer. After staining, cells were analyzed by using BD FACS AriaIII or BD FACS CantoII. FACS data were analyzed using FlowJo software (Tree Star, Inc.).

### BrdU staining

For BrdU incorporation assay, each mouse was injected with 1 mg of BrdU through i.p. twice at 12 h and 4 h before sacrifice. BM and spleen cells were harvested and processed according to the manufacturer’s protocol using APC-anti-BrdU and surface marker antibodies, including anti-CD11c, anti-IAb, anti-CD8, anti-CD11b, and anti-PDCA1 (BD, NJ). After staining, cells were analyzed by using BD FACS CantoII. FACS data were analyzed using FlowJo software (Tree Star, Inc.).

### Real-time PCR assay

Total RNAs were extracted from BM, spleen, purified DC, or 293T cells by using RNAiso Plus reagent (TAKARA Biotechnology Co.LTD, DALIAN). To detect miR-34a expression, total RNAs were reversed using MMLV reverse transcriptase with miR-34a specific RT primer 5′-CTCA ACTG GTGT CGTG GAGT CGGC AAT TCA GTT GAG ACA ACC AG-3′. The resultant cDNA was then used as template to perform real time PCR using a Roche real time PCR kit with specific PCR primers: F: 5′-ACA CTC CAG CTG GG TGG CAG TGT CTT AGC T -3′, R: 5′-CTC AAC TGG TGT CGT GGA-3′. To detect the expression of other genes, total RNAs were reversed using MMLV reverse transcriptase with Oligo (dT). Transcripts were quantified by real time PCR and normalized to the amount of GAPDH mRNA expression. The PCR primers are listed in Supplemental Table 1.

### Luciferase activity assay

The sequences predicted as the binding sites of miR-34a in Wnt1, and E2F2 mRNA, as well as their mutated oligos were synthesized, annealed and cloned downstream of CMV-driven firefly luciferase cassette in pMIR-REPORT vector (Ambion). All the sequences are shown in Supplemental Table 2. To validate miRNA targets, approximately 10^5^ 293T cells per well in a 24-well plate were transiently transfected with 0.3 μg of each firefly luciferase reporter construct, 0.1 μg of Renilla luciferase TK vector, and 0.6 μg of pMDH1-PGK-GFP-miR-34a. Renilla luciferase TK vector was used to normalize transfection efficiency. At 24 h after transfection, both firefly and Renilla luciferase activity were assayed (Promega). Normalized relative units represent firefly luciferase activity/Renilla luciferase activity.

### Northern blot analysis

Total RNAs were extracted from 293T, or pMDH1-PGK-GFP-mir-34a transfected 293T cells for the detection of expression of miR-34a using a highly sensitive microRNA Northern blot assay kit (SIgnosis, Inc., Sunnyvale, CA). Briefly, 15% TBE Urea-gel was pre-run at 60 V for approximately 30 min, and then 5 μg to 20 μg of RNA was loaded at 60 V. RNAs are transferred to a membrane at 60 V for 1 h, followed by UV cross linking for 10 min. The membrane is then hybridized with biotin-labeled miRNA probes and streptavidin-horseradish peroxidase (HRP) conjugate. The membrane is exposed using X-ray film after adding the substrate.18S and 28S RNA were used as loading reference.

### Western blot analysis

First, 293T cells transfected with pMSCV-EGFP-WNT1 or CD11c^+^ cells purified from miR-34a TG mice were lysed with RIPA lysis buffer (Beyotime Institute of Biotechology, Shanghai). Equal concentrations of protein were separated on a denaturing sodium dodecyl sulfate–10% polyacrylamide gel and then transferred to nitrocellulose by electro-blotting. Proteins were detected with a 1:1000 dilution of mouse anti-WNT1 (Affinity Biotechnology, OH) and a 1:5000 dilution of HRP-conjugated donkey anti-mouse antibodies (Cell Signaling technology, MA). GAPDH was used as loading reference. HRP were detected with Supersignal west Dura extended Duration substrate (Thermo Scientific, IL).

### T cell proliferation assay

Briefly, we firstly purified splenic CD4^+^ T cells from OVA-immunized WT mice, and co-cultured 2.0×10^5^ CD4 T cells with OVA primed (over-night) 2.0×10^4^ WT or miR-34a TG cDCs. Labeled these cells with CFSE or BrdU 24h later, and detected the proliferation activity of T cells at 72hrs later by flow cytometry.

### Statistical analysis

Statistical analysis was performed for all experiments using Student’s t-test. P values less than 0.05 were considered statistically significant. All data was shown as mean±S.E.M.

## SUPPLEMENTARY MATERIALS FIGURES AND TABLES


