# Supplementary material for: MiR-34a promotes DCs development and inhibits their function on T cell activation by targeting WNT1
Source: Oncotarget. 2017 Feb 9;8(10):17191–201. doi: 10.18632/oncotarget.15228 (PMC5370032; doi:10.18632/oncotarget.15228)
Supplement: Supplementary file 1 [file oncotarget-08-17191-s001.pdf]

MiR-34a promotes DCs development and inhibits their function on T cell activation by targeting WNT1

SUPPLEMENTARY FIGURES AND TABLES

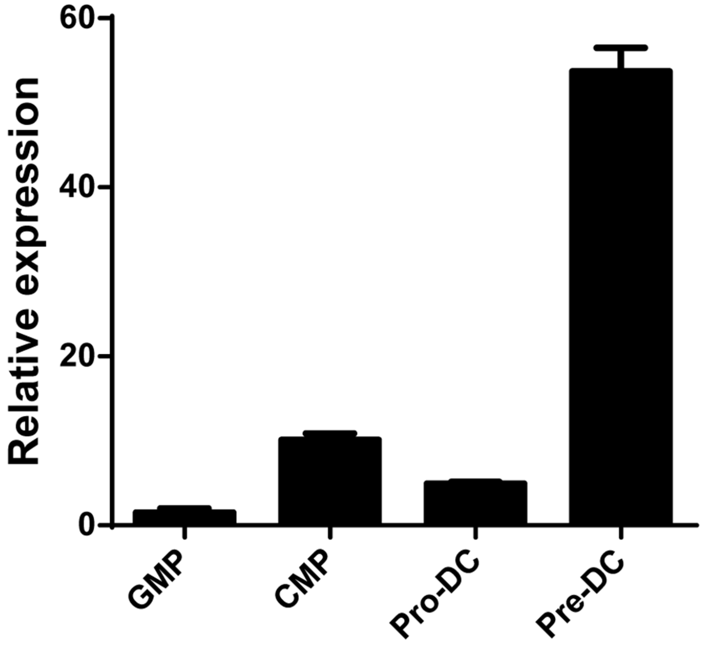

Supplementary Figure 1: The Expression of miR-34a in DC progenitors. We purified GMPs, CMPs, Pro-DCs and Pre-DCs from wild type BM cells and detected miR-34a expression level by qRT-PCR.

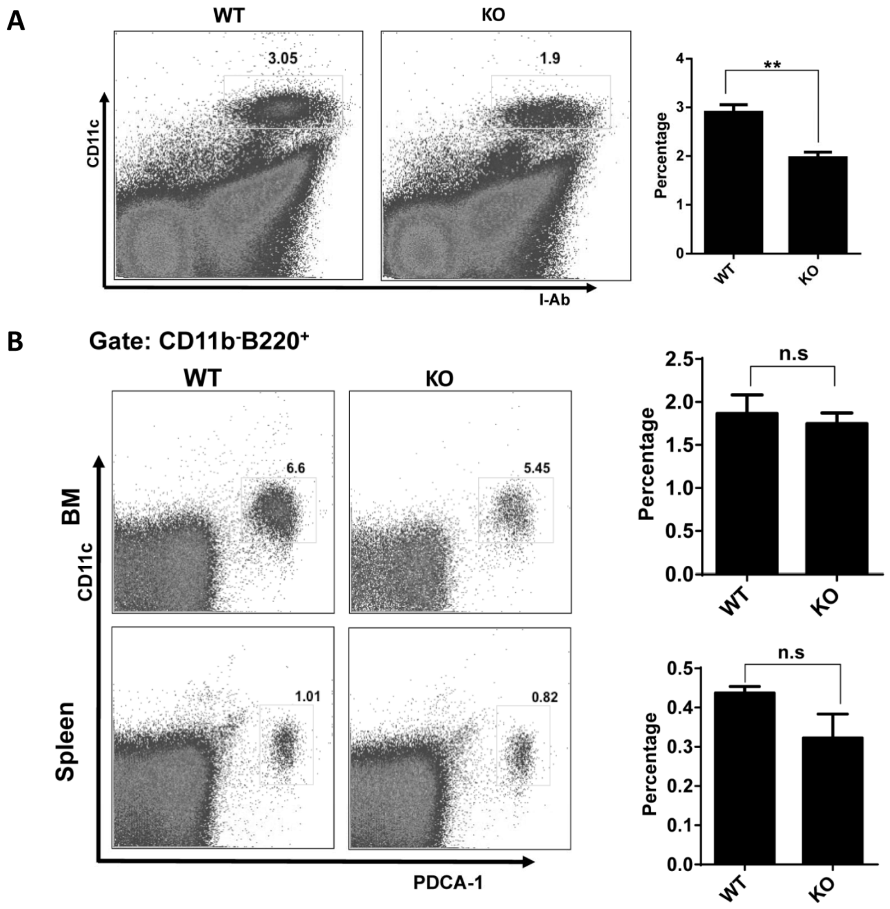

Supplementary Figure 2: The DC phenotype in miR-34a knockout mice. A. The phenotype of splenic cDCs in wild type (WT) or miR-34a knockout(KO) mice. **B.** The phenotype of pDCs from spleen or BM in WT or miR-34a KO mice. ** means $p<0.01$.

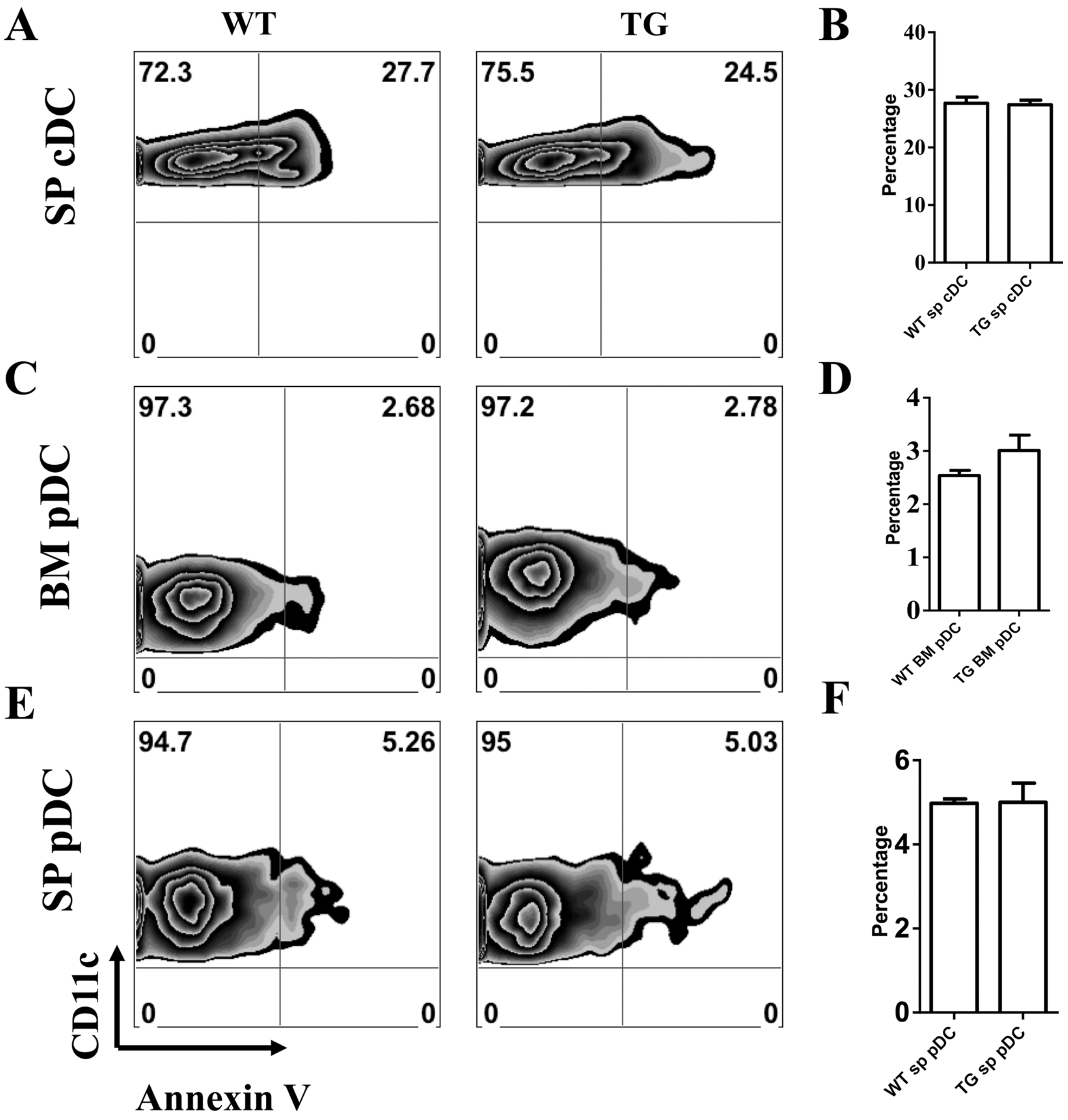

Supplementary Figure 3: Apoptosis of cDC and pDC in miR-34a transgenic mice. A, B. Apoptosis of cDC in spleen in WT and miR-34a TG mice. **C–F.** Apoptosis of pDC in BM (*C, D*) and spleen (*E, F*) in WT and miR-34a TG mice.

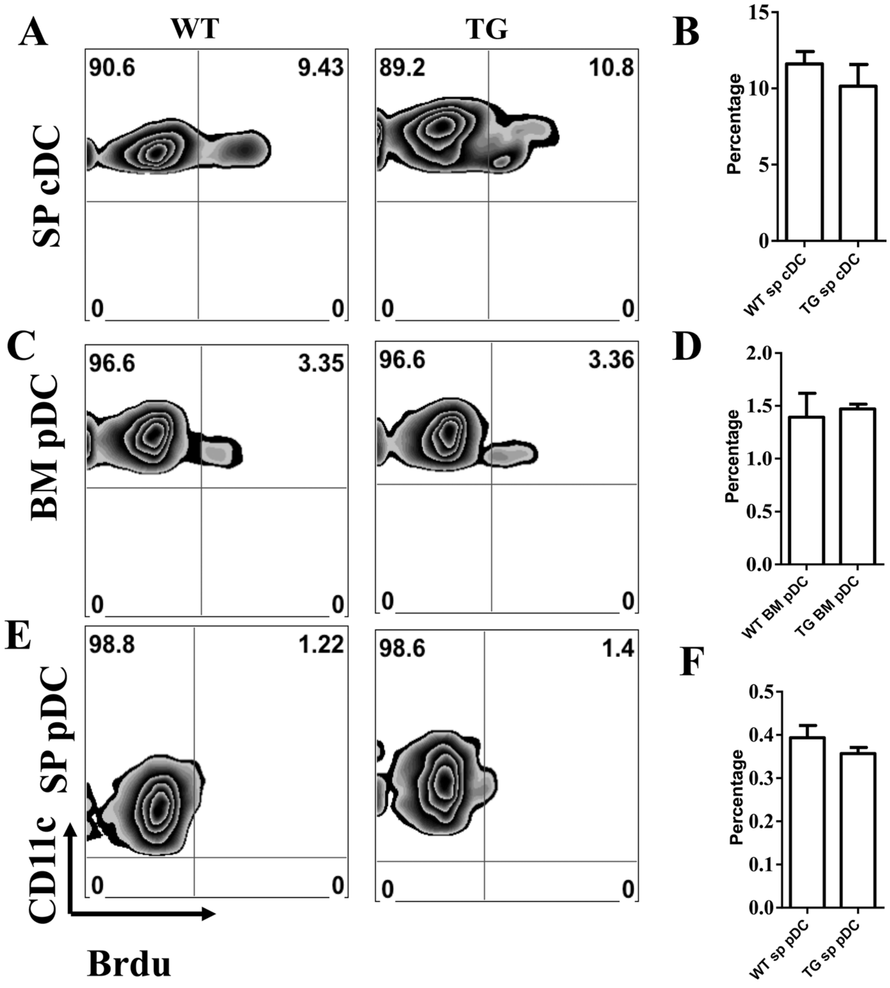

Supplementary Figure 4: Proliferation of cDCs and pDCs in miR-34a transgenic mice. A, B. Percentage of BrdU positive cells in cDCs in spleen in WT and miR-34a mice. **C–F.** Percentage of BrdU positive cells in pDC in BM (*C, D*) and spleen (*E, F*) in WT and miR-34a transgenic mice.

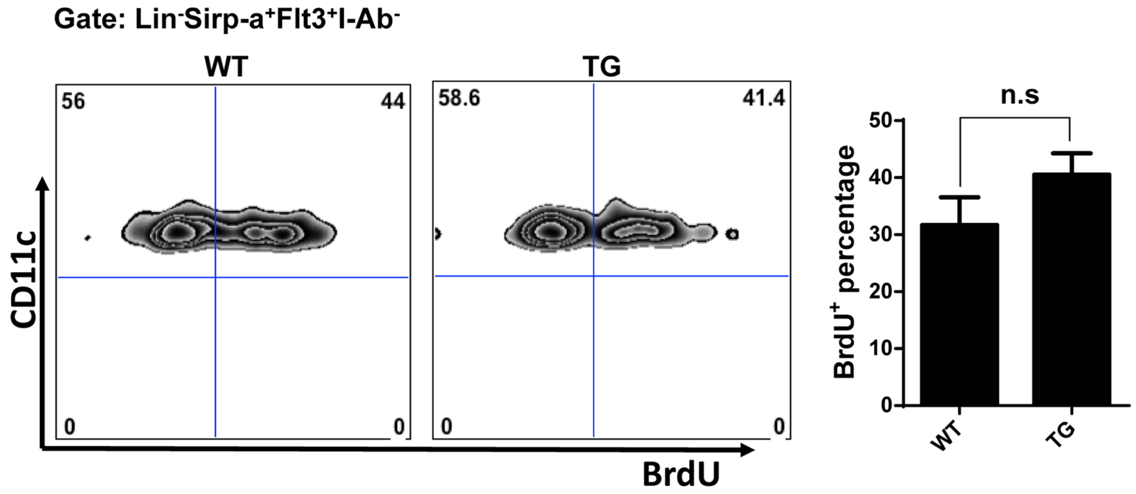

Supplementary Figure 5: miR-34a does not impair pre-DCs proliferation *in vivo*. All group of mice were subject to BrdU (1 mg/mice) injection (i.p.), and the proliferation of splenic pDCs were examined by flow cytometry.

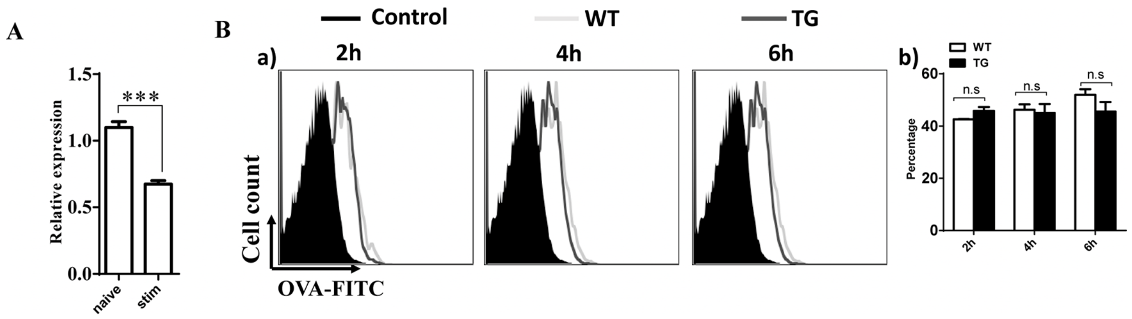

Supplementary Figure 6: Overexpression of miR-34a in DCs does not affect function of antigen uptake of DCs. A. Expression of miR-34a in naive and antigen-stimulated DCs. **B.** OVA-FITC antigen uptake by DC from wild type (WT, black) and miR-34a transgenic mice (TG, red), non-addition of OVA-FITC as control (gray). ***means p<0.001.

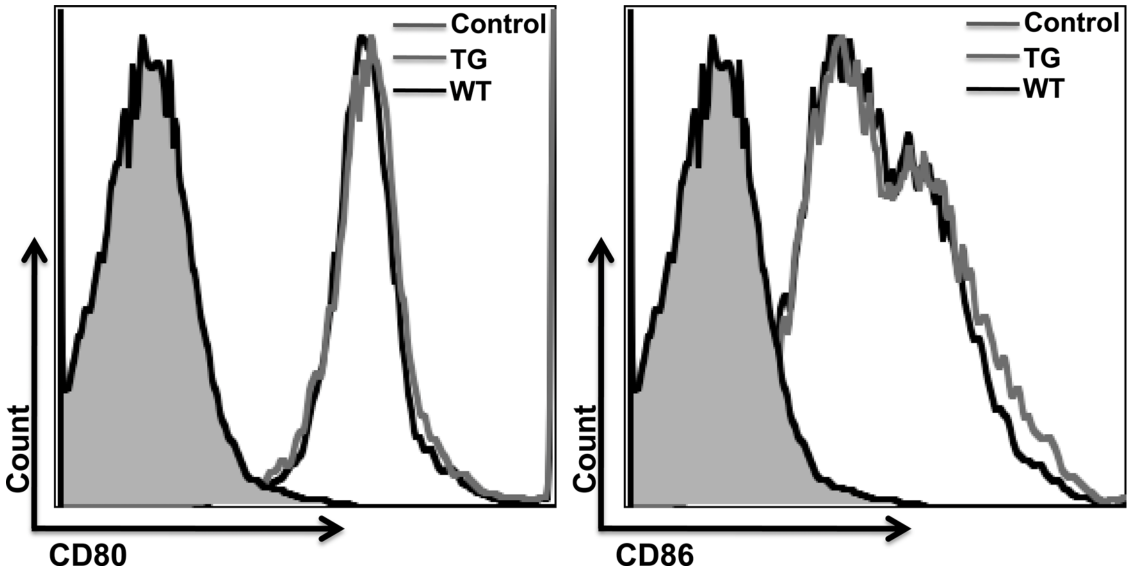

Supplementary Figure 7: The Expressions of CD80, CD86 in cDCs in WT (black) and miR-34a transgenic mice (TG, red).

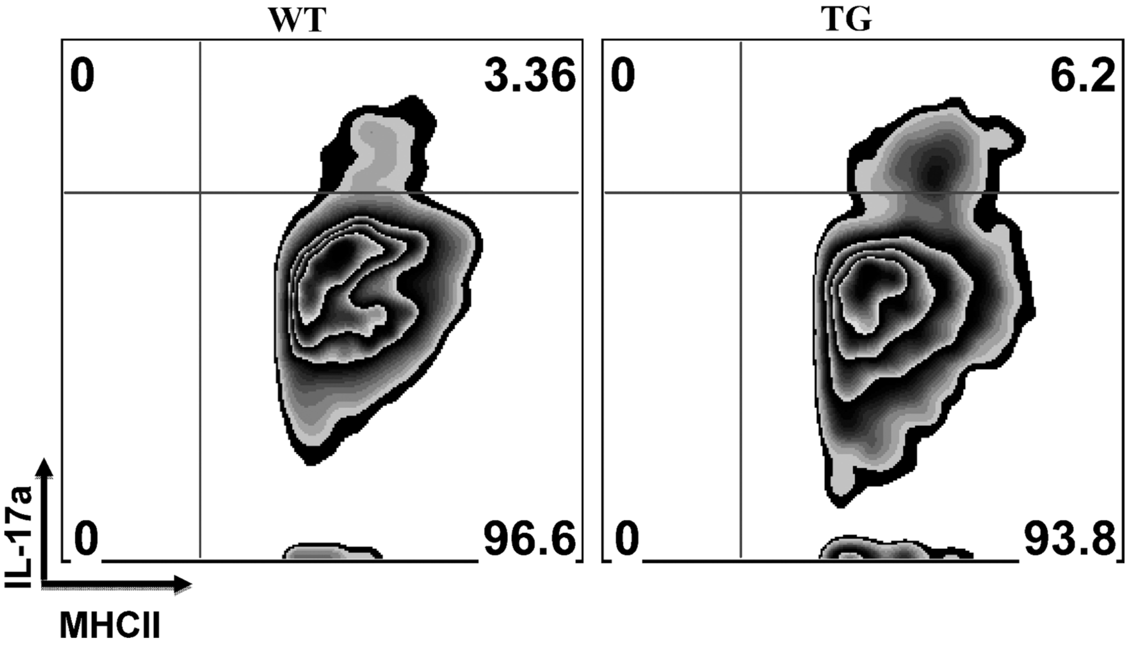

Supplementary Figure 8: The Expression of IL-17a in miR-34a overexpressed cDC. Spleen cells were stained with anti-CD11c, anti-IAb and intra-cellular stained with anti-IL-17a antibodies. Cells were analyzed with FACS, and gated on $CD11c^{high}IAb^{+}$. Percentage of $IL\text{-}17a^{+}IAb^{+}$ cells from WT and miR-34a TG (TG) were shown.

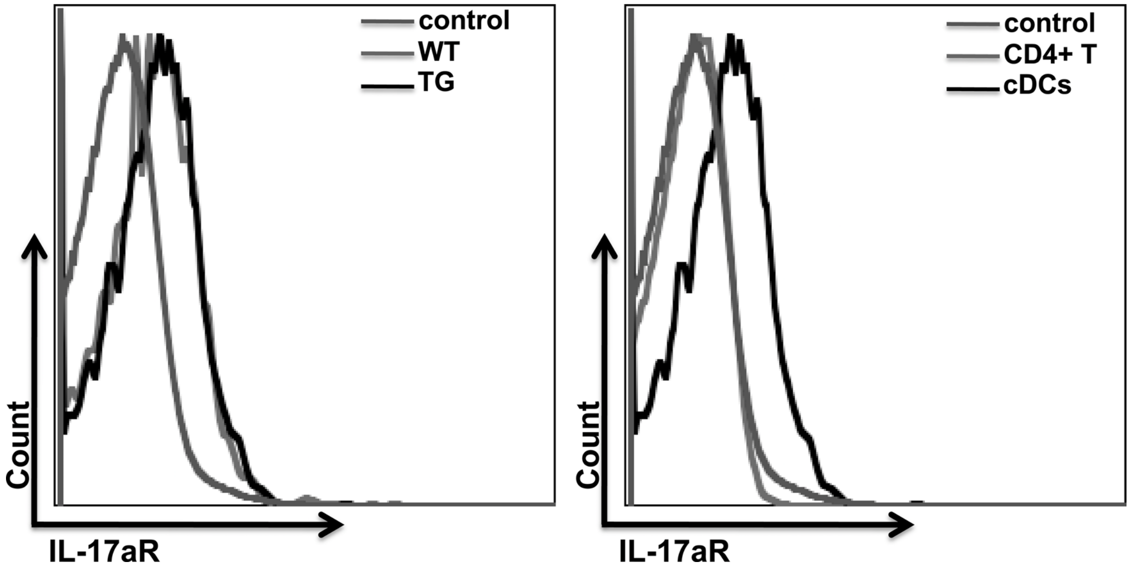

Supplementary Figure 9: Expression of IL-17 receptor in cDCs from WT or Tg mice. Spleen cells were stained with anti-CD4 or anti-CD11c/IAb and anti-IL-17aR antibodies. Expressions of IL-17aR in $CD4^+$ T cells and that in cDC merged from wild type mice (left), and IL-17aR in WT cDC (WT) and that in miR-34a TG cDC (TG) (right).

Supplementary Table 1: Some primers used for real time PCR and cloning

gene	primer pairs	sequence
TNF-a	Forward	5' CCACACCGTCAGCCGATTTG 3'
	Reverse	5' CACCCATTCCCTTCACAGAGC 3'
IL-6	Forward	5' TGGGAAATCGTGGAAATGAG 3'
	Reverse	5' GAAGGACTCTGGCTTTGTCTT 3'
IL-10	Forward	5' CAAGGCAGTGGAGCAGGTGAA 3'
	Reverse	5' CGGAGAGAGGTACAAACGAGGTT 3'
IL-12	Forward	5' AACCAGACCCGCCCAAGAAC 3'
	Reverse	5' GATCCTGAGCTTGCACGCAGA 3'
IFN-γ	Forward	5' GCCACGGCACAGTCATTGAAA 3'
	Reverse	5' TTTCGCCTTGCTGTTGCTGA 3'
IL-17A	Forward	5' CCTCAAAGCTCAGCGTGTCC 3'
	Reverse	5' GAGCTCACTTTTGCGCCAAG 3'
TGF-beta	Forward	5' TGGGCACCATCCATGACAT 3'
	Reverse	5' TCTTCTCTGTGGAGCTGAAGCA 3'
Wnt1(clone)	Forward	5' CGCCGGAATTAGATCTCGCAACC ACAGTCGTCAGAAC 3'
	Reverse	5' ATTCGTTAACCTCGATCATAGACA CTCGTGCAGAAC 3'
E2F2	Forward	5'AGTTGCTCCCTGAGCTTCAAG3'
	Reverse	5'TGACCGCAATCACTGTCTGCT3'
GAPDH	Forward	5'AGAAACCTGCCAAGTATGATGACA3'
	Reverse	5'GGAAGAGTGGGAGTTGCTGTTG3'
WNT1	Forward	5' CGCTCTCTTCCAGTTCTCAGACAC 3'
	Reverse	5' CAGGATGGCAAAAGGGTTCG 3'
TCF1	Forward	5'AGCACACTTCGCAGAGACTTT3'
	Reverse	5'GTGGACTGCTGAAATGTTCG3'
RORrt	Forward	5'CCGCTGAGAGGGCTTCAC3'
	Reverse	5'TGCAGGAGTAGGCCACATTACA3'

Supplementary Table 2: Primers used for construction of pMir-Report plasmids

Primer name	Sequence
E2F2-LU-F	5' CTAGTCAGGTGGCCATGTTCAGGGAAAGGGCTCACTGCCTCCTTCTGAGA 3'
E2F2-LU-R	5' AGCTTCTCAGAAGGAGGCAGTGAGCCCTTTCCCTGAACATGGCCACCTGA 3'
WNT1-LU-F	5' CTAGTCCCTCTTTGAGGGAGACTCCTTTTGCACTGCCCCCCAATTTGGA 3'
WNT1-LU-R	5' AGCTTCCAAATTGGGGGGCAGTGCAAAAGGAGTCTCCCTCAAAGAGGGA 3'
m-E2F2-LU-F	5' CTAGT CAGGTGGCCATGTTCAGGGAAAGGGCTCAGACGGTCCTTCTGAGA 3'
m-E2F2-LU-R	5' AGCTTCTCAGAAGGACCGTCTGAGCCCTTTCCCTGAACATGGCCACCTGA 3'
m-WNT1-LU-F	5' CTAGTCCCTCTTTGAGGGAGACTCCTTTTGCAGACGGCCCCAATTTGGA 3'
m-WNT1-LU-R	5' AGCTTCCAAATTGGGGCCGTCTGCAAAAGGAGTCTCCCTCAAAGAGGGA 3'
